# Identifying CTH and MAP1LC3B as ferroptosis biomarkers for prognostic indication in gastric cancer decoding

**DOI:** 10.1038/s41598-024-54837-9

**Published:** 2024-02-22

**Authors:** Haishun Qu, Yunxiao Liang, Quan Guo, Ling Lu, Yanwei Yang, Weicheng Xu, Yitian Zhang, Yijue Qin

**Affiliations:** Guangxi Academy of Medical Sciences, People’s Hospital of Guangxi Zhuang Autonomous Region, Nanning, China

**Keywords:** Gastric cancer, Ferroptosis, Prognostic model, Immune cell infiltration, Immunohistochemistry, Cancer microenvironment, Tumour biomarkers, Gastric cancer

## Abstract

Gastric cancer (GC), known for its high incidence and poor prognosis, urgently necessitates the identification of reliable prognostic biomarkers to enhance patient outcomes. We scrutinized data from 375 GC patients alongside 32 non-cancer controls, sourced from the TCGA database. A univariate Cox Proportional Hazards Model (COX) regression was employed to evaluate expressions of ferroptosis-related genes. This was followed by the application of Least Absolute Shrinkage and Selection Operator (LASSO) and multivariate COX regression for the development of prognostic models. The composition of immune cell subtypes was quantified utilizing CIBERSORT, with their distribution in GC versus control samples being comparatively analyzed. Furthermore, the correlation between the expressions of Cystathionine Gamma-Lyase (CTH) and Microtubule Associated Protein 1 Light Chain 3 Beta (MAP1LC3B) and the abundance of immune cell subtypes was explored. Our bioinformatics findings underwent validation through immunohistochemical analysis. Our prognostic models integrated CTH and MAP1LC3B. Survival analysis indicated that patients categorized as high-risk, as defined by the model, exhibited significantly lower survival rates compared to their low-risk counterparts. Notably, CTH expression inversely correlated with monocyte levels, while MAP1LC3B expression showed an inverse relationship with the abundance of M2 macrophages. Immunohistochemical validation corroborated lower expressions of CTH and MAP1LC3B in GC tissues relative to control samples, in concordance with our bioinformatics predictions. Our study suggests that the dysregulation of CTH, MAP1LC3B, and the accompanying monocyte-macrophage dynamics could be pivotal in the prognosis of GC. These elements present potential targets for prognostic assessment and therapeutic intervention.

## Introduction

Gastric cancer (GC) ranks among the most prevalent malignancies globally, often diagnosed in advanced stages due to initially subtle symptoms and low regular screening rates. Recent years have seen significant strides in systemic therapies for GC, spanning chemotherapy, targeted treatments, and immunotherapies. This has led to a paradigm shift in managing resectable and metastatic GC, with a growing focus on individualized treatment strategies based on molecular biomarkers^1^. Gastric cancer (GC) stands as a significant contributor to the rising incidence and mortality rates of cancer globally^2^. Ranking as the third leading cause of cancer-related deaths worldwide, over 95% of gastric cancers manifest as adenocarcinomas. A primary factor in the grim prognosis associated with gastric cancer is its tendency for late-stage diagnosis, often missing the optimal window for effective treatment and intervention^3^. Surgery, a cornerstone in gastric cancer treatment, is vital across all stages of the disease. In cases of low-risk and early-stage lymph node metastasis, a combination of surgical and endoscopic treatments has been shown to yield more favorable outcomes^4^. With the advancement of bioinformatics, an increasing array of methods is being applied in the field of medical research, providing us with many novel ways and approaches to understand diseases^5–10^. Given the low survival rates and challenges in early diagnosis of gastric cancer, there is an acute need to identify prognostic biomarkers. These biomarkers are crucial for the early detection of gastric cancer and for predicting patient prognosis.

Ferroptosis represents a distinctive non-apoptotic form of cell death, predominantly triggered by uncontrolled accumulation of peroxisomal phospholipids and further exacerbated by iron-catalyzed oxygen radical production^11^. This death pathway has been observed across various organisms, including mammals, serving a dual role: it combats pathogens and also effectively eliminates malignant tumor cells. Intriguingly, tumor cells have developed adaptive metabolic strategies to counteract this mechanism. They inhibit ferroptosis by modulating ferroptinase activity, thereby safeguarding themselves, and disrupt redox homeostasis while inducing iron deposition to alter tumor energy metabolism^12^. A notable gap in our understanding is the mechanism underlying ferroptin induction in tumor cells. Recent research by Peng Liao et al. sheds light on this area, demonstrating that interferon (IFN) γ from T cells, in concert with arachidonic acid (AA), can initiate the immune ferroptosis pathway, leading to tumor cell demise. This process is pivotal in CD8 + T cell (CTL)-mediated eradication of tumor cells^13^. Furthermore, the relevance of ferroptosis extends to cancer treatment resistance, with its induction shown to potentially reverse drug resistance in tumor cells^14^. While the ferroptosis pathway is crucial in cancer progression and intimately linked with drug resistance in tumors, research specifically addressing ferroptosis and drug sensitivity in gastric cancer remains notably scarce.

Immune dysregulation within the tumor microenvironment is increasingly recognized as a pivotal factor in cancer progression^15^. Myeloid-derived suppressor cells (MDSCs), which encompass activated monocytes and neutrophils in pathological states, exhibit potent immunosuppressive activities. These cells play a significant role in the modulation of immune responses across various pathological contexts and are strongly linked to adverse cancer outcomes^16^. Mononuclear phagocytes (MNP), including monocytes, macrophages (MoMac), and dendritic cells, serve critical functions in immunomodulation, antimicrobial defense, and maintaining bodily homeostasis^17^. Notably, microbiota signaling can steer the differentiation of mononuclear phagocytes in the tumor microenvironment towards immunostimulatory monocytes and dendritic cells (DCs)^18^. The immune response to cancer is notably heterogeneous. Tumors deficient in mismatch repair (MMRd) demonstrate more pronounced anti-tumor immune characteristics compared to mismatch repair-proficient (MMRp) tumors, an observation closely linked to alterations in the immune microenvironment^19^.

Our study is designed to meticulously screen for genes that intersect between gastric cancer (GC) and ferroptosis using advanced bioinformatics analyses. The primary aim is to construct a prognostic model for GC and to identify biomarkers that can aid in its early diagnosis and prognosis. To this end, we employed CIBERSORT to analyze alterations in immune cell populations within GC, with a particular focus on elucidating the role of monocytes in the disease's pathophysiology.

## Materials and methods

### Data acquisition and preliminary processing

Gene expression data for gastric cancer (GC) and corresponding normal controls were procured from The Cancer Genome Atlas (TCGA)^20^. This dataset included comprehensive transcriptome profiling data. We performed normalization of the data from both the experimental (GC) and control (normal) groups using the R programming language (× 64, version 4.1.3) to facilitate analysis on a uniform scale. For statistical analysis, IBM SPSS Statistics 26 was employed, with the exception of the quantification of positive immunohistochemical images, which was conducted using R. Additionally, we downloaded clinical data of GC patients, encompassing ID numbers, survival status, survival duration, and other pertinent information.

### Extraction and analysis of ferroptosis genes

To delve into the role of Ferroptosis genes in GC, we retrieved a Ferroptosis gene set from the Gene Set Enrichment Analysis (GSEA) database. Using the "limma" package in R, we first isolated Ferroptosis gene expressions from the GC genome. A differential expression analysis between experimental and control groups was then conducted using the Wilcoxon rank sum test. Visual representations of these findings were created: a heat map of Ferroptosis genes using the "pheatmap" package, and a violin plot comparing expressions in GC and control groups using "ggplot2". Additionally, we calculated and depicted correlations among Ferroptosis genes through correlation heat maps, utilizing both the "corrplot" and "circlize" packages.

### Functional enrichment and pathway analysis in gastric cancer cases

In our study focusing on gastric cancer (GC) cases, we conducted an in-depth analysis of the gene ontology (GO) and KEGG pathway enrichment of Ferroptosis genes. This was achieved by utilizing a combination of sophisticated packages in R, which included "clusterProfiler", "org.Hs.eg.db", "enrichplot", "ggplot2", and "GOplot". These tools were integral in executing both the GO enrichment analysis and the KEGG pathway enrichment analysis.

The "clusterProfiler" package was particularly crucial, as it facilitated the statistical analysis and visualization of the functional profiles of genes and gene clusters. This allowed us to delve into the biological functions, cellular components, and molecular processes in which the Ferroptosis genes are involved. Meanwhile, the "org.Hs.eg.db" package provided essential annotations of human genes, which served as a reference for our analyses.

For visual representation and enhanced interpretability of the results, we employed the "enrichplot" and "ggplot2" packages. The "enrichplot" package was used to generate intuitive visualizations of the enrichment results, making it easier to interpret complex gene interactions and pathways. The "ggplot2" package further aided in creating high-quality, customizable graphics for our data.

In addition to these analyses, we also performed Gene Set Variation Analysis (GSVA) enrichment using a combination of "limma", "org.Hs.eg.db", "clusterProfiler", and "enrichplot" packages. The "limma" package, known for its capabilities in analyzing gene expression data, especially from microarray and RNA-seq experiments, was instrumental in identifying differentially expressed genes and pathways in GC. This comprehensive approach allowed us to gain deeper insights into the gene functions and pathways involved in gastric cancer, enhancing our understanding of the disease at the molecular level.

### Prognostic model construction for GC ferroptosis

To elucidate the relationship between Ferroptosis genes and gastric cancer (GC) prognosis, we developed a logistic regression model through a structured approach. The first step involved conducting univariate COX regression analysis^21^ to correlate each Ferroptosis gene with the survival status and time of GC patients. This initial phase was crucial for establishing a foundational understanding of the individual genes' impacts, with a significance threshold set at p < 0.05.

Following this, we progressed to a secondary screening using Least Absolute Shrinkage and Selection Operator (LASSO) regression analysis^22^. LASSO's ability to handle models with numerous predictors made it an ideal choice for refining our analysis, effectively selecting the most relevant genes while controlling for overfitting.

The culmination of our modeling process was the application of multivariate COX regression analysis^23^. This allowed us to analyze multiple genes concurrently with GC survival status and time. Through this, we identified critical genes, namely CTH and MAP1LC3B, and calculated their risk scores. These scores were instrumental in constructing our final model.

Patients were then categorized into high and low-risk groups based on their median risk scores. This classification enabled a more nuanced understanding of the prognostic implications of Ferroptosis genes in GC, shedding light on their potential roles in patient outcomes and disease progression.

### Gene expression and risk assessment in different risk groups

We evaluated the expression of CTH and MAP1LC3B in high and low-risk gastric cancer (GC) groups using the "ggplot2" package in R. This was visualized as violin plots, effectively showing the distribution and differences in gene expression between the two groups. Additionally, we created a heat map using the "pheatmap" package to illustrate the expression patterns of these genes across our GC patient cohort. This heat map was organized from low to high risk, incorporating survival information as a dot plot, thereby providing a clear, visual representation of the relationship between gene expression, risk level, and patient survival outcomes.

### Survival analysis

In our survival analysis of gastric cancer (GC) cases, we utilized a two-step approach. First, we categorized patients into high and low expression groups based on the median expression of key genes. For these groups, we calculated survival curves to evaluate the impact of gene expression on patient survival. These curves were visually represented, allowing for a clear comparison between the high and low expression groups.

Subsequently, following the development of our prognostic model, we assigned risk scores to each patient. Based on these scores, patients were again divided into high and low-risk groups. We calculated and visualized the survival curves for these groups as well. This additional step provided us with a deeper understanding of how our prognostic model's risk scores correlated with patient outcomes, offering a more comprehensive analysis of survival in GC cases.

### Testing and prognosis prediction of GC prognostic models

To assess our gastric cancer (GC) prognostic model's accuracy, we used the receiver operating characteristic curve (ROC) method. This allowed us to evaluate the model's ability to distinguish between patient outcomes effectively. Additionally, we created column line plots that included variables like age, sex, and risk levels to enhance the prediction of GC patient prognosis. We also constructed calibration plots to compare the model's predictions with actual outcomes, ensuring the model's reliability in a clinical setting.

### Drug sensitivity analysis

In our study, to identify potential drugs sensitive to gastric cancer (GC) treatment targeting the genes CTH and MAP1LC3B, we utilized the CellMiner™ database^17^. This comprehensive database encompasses data from 60 cancer cell lines developed by the National Cancer Institute (NCI). It provides extensive information on gene sensitivity, covering a vast array of 22,379 genes and 20,503 compounds. By analyzing the interaction between CTH and MAP1LC3B genes and these compounds, we aimed to pinpoint drugs that could be particularly effective in treating GC, leveraging the database's robust predictive capabilities in gene-drug sensitivity correlations.

### Immune cell composition and correlation analysis

To investigate the differences in immune cell composition between the experimental and control groups in our study, we employed the CIBERSORT algorithm^24^. CIBERSORT is a sophisticated tool that quantifies the abundance of specific immune cell types within complex tissues from their gene expression profiles. By applying this method, we were able to accurately estimate the proportions of various immune cells in both groups.

Further, we calculated the correlations between different immune cell subtypes. To visualize these correlations, we utilized two R packages: "corrplot" and "circlize". The "corrplot" package was used to create correlation matrices, offering a clear and intuitive representation of the relationships between the different immune cell types. The "circlize" package was employed to generate circular visualization plots, providing a comprehensive and aesthetically appealing way to display the intricate network of correlations among the immune cells. These visualizations were crucial for a deeper understanding of the immune landscape in our experimental setup.

### Immune cell differential and correlation analysis

For our study on gastric cancer (GC), we analyzed the differential expression and correlation of CTH and MAP1LC3B with immune cells in GC and normal groups. We used the "limma" package for identifying differential expressions and the "vioplot" package for creating violin plots to visually represent these differences. Additionally, we employed "ggplot2", "limma", "ggpubr", and "ggExtra" for detailed correlation visualizations, showcasing the interactions between these genes and immune cells in both groups.

### Immunohistochemistry and hematoxylin–eosin staining

In this crucial part of our study, we aimed to validate the bioinformatics analysis through detailed immunohistochemical examination of CTH and MAP1LC3B, which were pivotal genes identified in our model. The tissues employed for this purpose were meticulously collected from intraoperative excisions at the Guangxi Zhuang Autonomous Region People's Hospital. This procedural component received ethical clearance from the Ethics Committee of the Guangxi Academy of Medical Sciences and the People's Hospital of Guangxi Zhuang Autonomous Region. Furthermore, due to the anonymity of the patient samples, the ethics review committee granted an exemption from the usual informed consent requirement.

For the immunohistochemical staining, we carefully selected specific antibodies from Proteintech, utilizing item numbers 12217-1-AP for CTH and 14600-1-AP for MAP1LC3B. This choice was driven by the need for high specificity and sensitivity in detecting our target proteins. The tissue preparation process was comprehensive, involving formalin fixation to preserve cellular and tissue architecture, followed by sequential dehydration and waxing for embedding. Precise sectioning was performed to obtain optimal tissue slices, which were then dewaxed and rehydrated. This was followed by antigen retrieval, a critical step to unmask the antigen sites, and blocking of endogenous peroxidase to prevent non-specific staining.

The tissues were then incubated with the primary antibodies specific to CTH and MAP1LC3B, followed by appropriate secondary antibodies. This step was crucial for the visualization of the antigen–antibody complexes. After incubation, the slides underwent color development and restaining to enhance contrast, concluding with careful slide mounting for microscopic examination.

The stained slides were meticulously examined under an inverted microscope. This high-resolution imaging allowed for detailed observation of the staining patterns, indicative of the presence and localization of the target proteins. Quantitative analysis of the staining intensity and distribution was conducted using Image J software, a powerful tool for image analysis in biological studies. The quantified data were then statistically analyzed using IBM SPSS Statistics 26, employing independent sample t-tests to assess the significance of our findings. The results were graphically represented using GraphPad Prism 8, providing a clear and visually appealing presentation of the data.

In addition to immunohistochemistry, we also conducted Hematoxylin–Eosin (HE) staining, a classic histological technique that provides essential insights into tissue morphology and pathology. This process involved several meticulous steps, starting with tissue hydration and subsequent staining with hematoxylin to highlight cell nuclei. This was followed by differentiation, a step to remove excess stain, and counterstaining with eosin, which colors the cytoplasm and other cellular components. The tissue sections were then dehydrated, air-dried, and sealed for preservation.

HE-stained slides were also examined under an inverted microscope, allowing us to observe and document the histopathological features of GC and control tissues. This analysis was vital in corroborating our immunohistochemical findings and adding another layer of validation to our bioinformatics results, thereby enhancing the overall robustness and credibility of our study.

### Ethical disclosure

This study was approved by the Ethics Review Committee of the People's Hospital of Guangxi Zhuang Autonomous Region and was in accordance with the Declaration of Helsinki of the World Medical Association.

## Results

### Data download and preliminary processing

**Figure 1:**
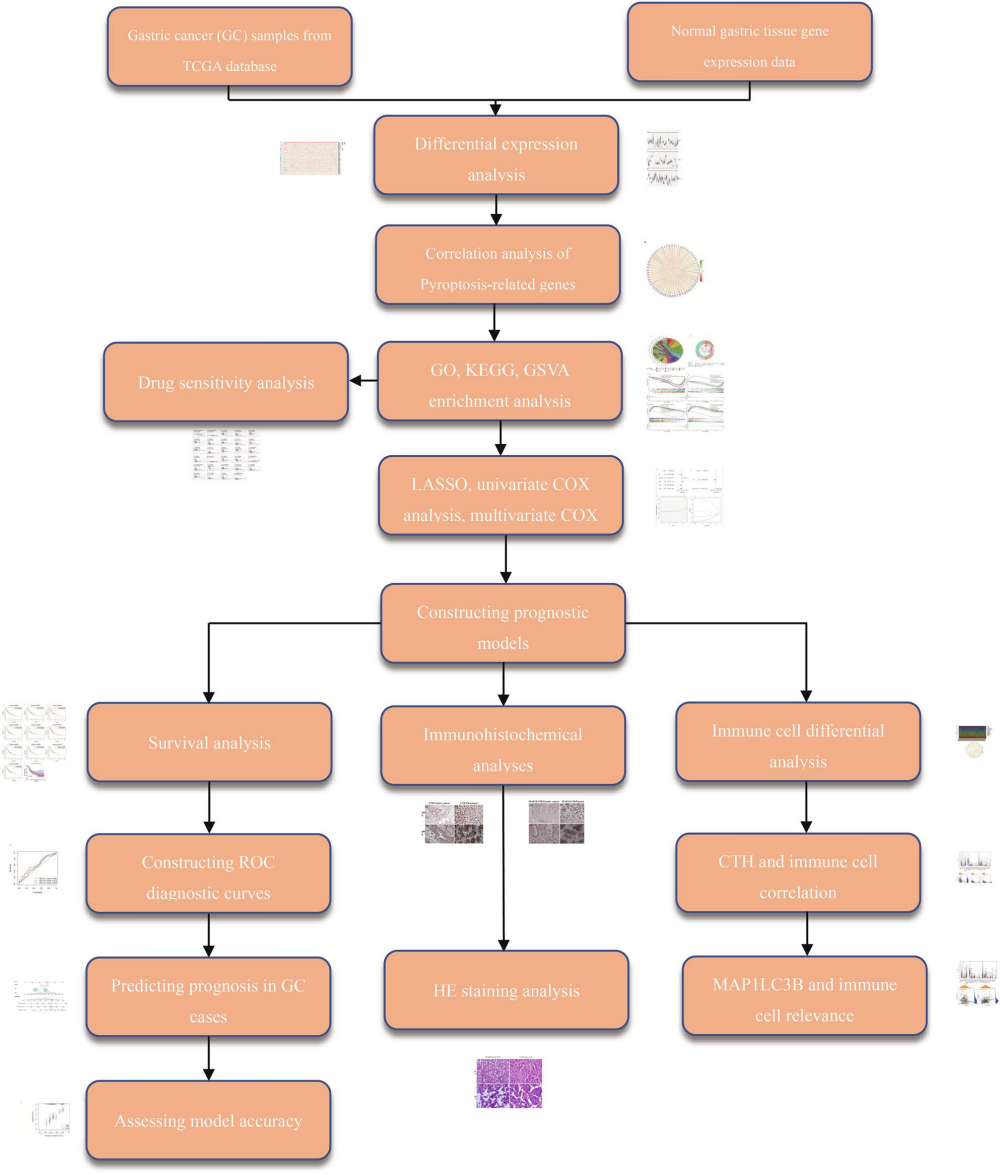
Workflow diagram. Here, we present the workflow diagram of the work conducted in this study.

We procured transcriptomic data for a cohort of 375 gastric cancer (GC) cases alongside 32 normal control samples from The Cancer Genome Atlas (TCGA) database. This comprehensive dataset underwent a meticulous preprocessing phase, where raw data were converted into recognizable gene symbols. The normalization process was employed to ensure data comparability across samples, adhering to robust statistical methods. Post-normalization, we assembled a dataset encompassing a total of 411 cases, each accompanied by complete clinical information pertinent to GC. This dataset formed the foundation for our subsequent analyses, providing a rich repository of gene expressions and clinical correlations. We have placed the workflow diagram of the entire research process in Fig. 1.

### Ferroptosis gene extraction and analysis

**Figure 2:**
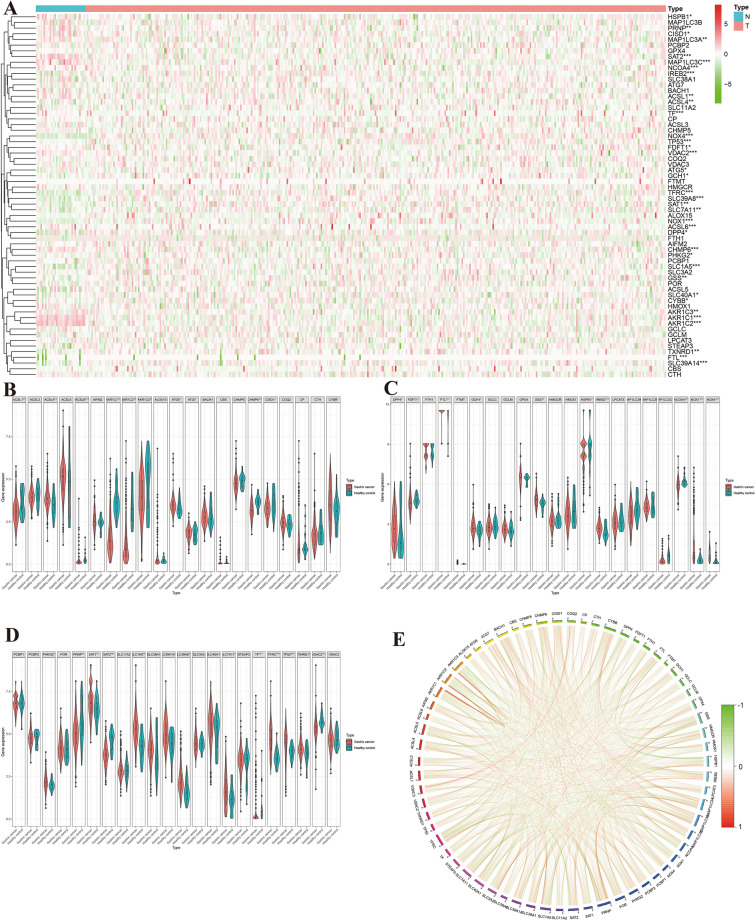
Differential expression of ferroptosis-associated genes in gastric cancer. Figure (**A**) illustrates the heat map of differential gene expression, where red squares denote high expression genes, and green squares indicate low expression genes. Figures (**B**–**D**) depict violin plots of differential gene expression, with red representing expression in GC and green in controls. Figure (**E**) shows the Ferroptosis gene correlation plot; red lines suggest synergistic high expression, and green lines indicate synergistic low expression.

In our expansive analysis encompassing 39,953 genes, we meticulously identified 64 genes integral to the ferroptosis process. Utilizing a rigorous differential expression analysis framework, we discerned 36 of these ferroptosis-related genes to be significantly differentially expressed between the gastric cancer (GC) cases and control samples. This key finding is depicted in Fig. 2A–D, providing a visual representation of the expression disparities. Further enhancing our understanding, a comprehensive co-expression analysis was conducted, which unveiled patterns of both concurrent low (co-low) and high (co-high) expressions among these ferroptosis genes, as elaborately illustrated in Fig. 2E. These results not only augment our knowledge of the ferroptosis landscape in GC but also pave the way for deeper insights into the molecular intricacies underpinning this disease.

### Gene function and pathway analysis results

**Figure 3:**
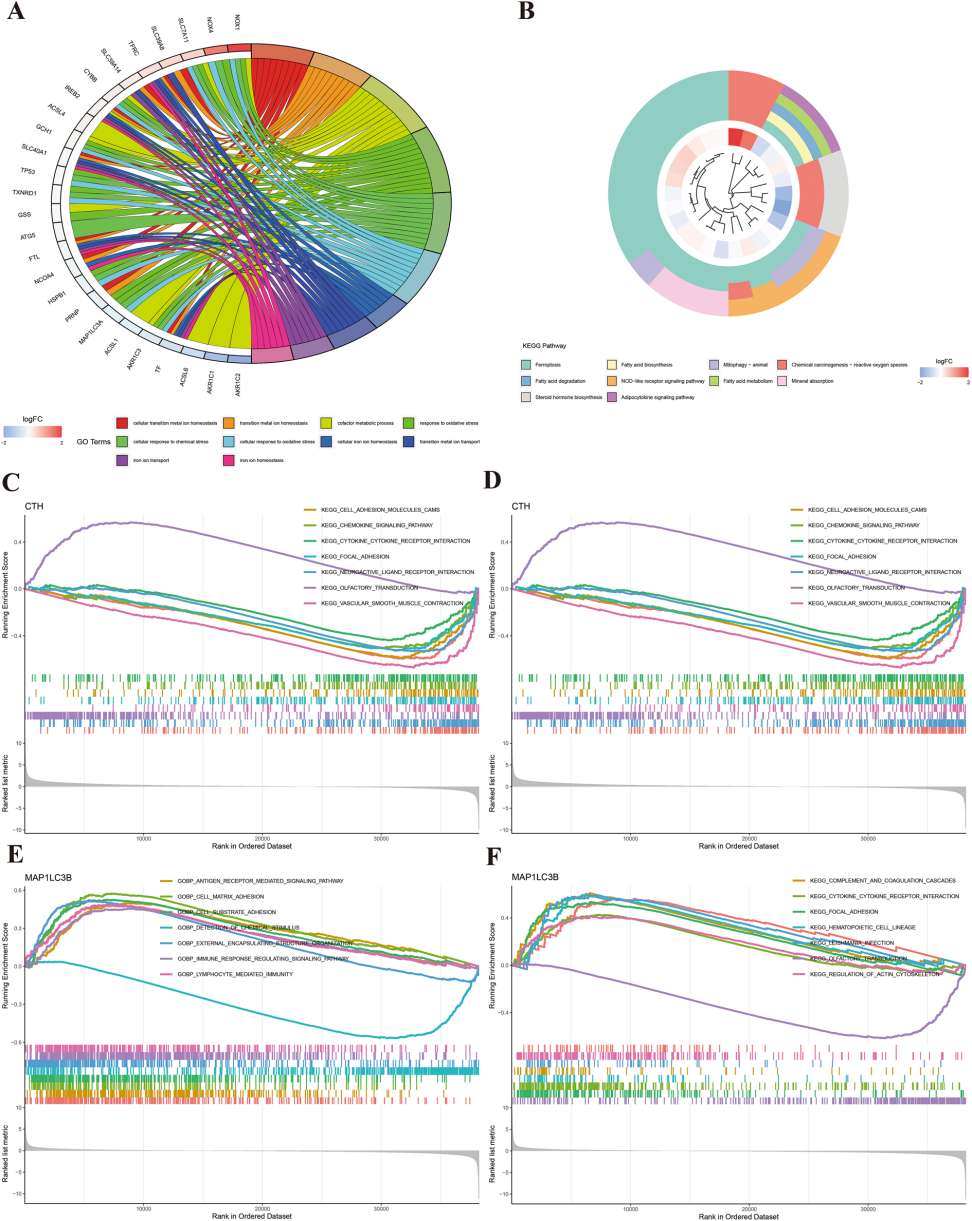
Gene enrichment analysis plots. Figures (**A**) and (**B**) demonstrate the results of GO enrichment analysis and KEGG pathway enrichment analysis for ferroptosis genes. Figures (**C**) and (**D**) represent GSVA analysis of CTH, while Figures E and F showcase GSVA enrichment analysis results for MAP1LC3B.

Delving into the functional landscape of the identified genes, we conducted a Gene Ontology (GO) enrichment analysis. The results, as illustrated in Fig. 3A, predominantly revealed a significant enrichment in processes related to cellular metal ion homeostasis and transport. This finding underscores the intricate role of metal ions in the pathophysiology of gastric cancer.

Furthermore, our exploration extended to the Kyoto Encyclopedia of Genes and Genomes (KEGG) pathway^25–28^ analysis. As detailed in Fig. 3B, this analysis brought to light an enrichment of the ferroptosis pathway and several related pathways, reinforcing the potential mechanistic link between ferroptosis and gastric cancer development.

To gain deeper insights into the specific roles of key genes, Gene Set Variation Analysis (GSVA) was employed. This analysis highlighted distinct enrichment patterns for Cystathionine Gamma-Lyase (CTH) and Microtubule Associated Protein 1 Light Chain 3 Beta (MAP1LC3B), as shown in Fig. 3C–F, respectively. The differential enrichment patterns observed for CTH and MAP1LC3B suggest their unique contributions to the molecular dynamics of gastric cancer, potentially offering avenues for targeted therapeutic strategies.

### Construction of GC ferroptosis prognostic model

**Figure 4:**
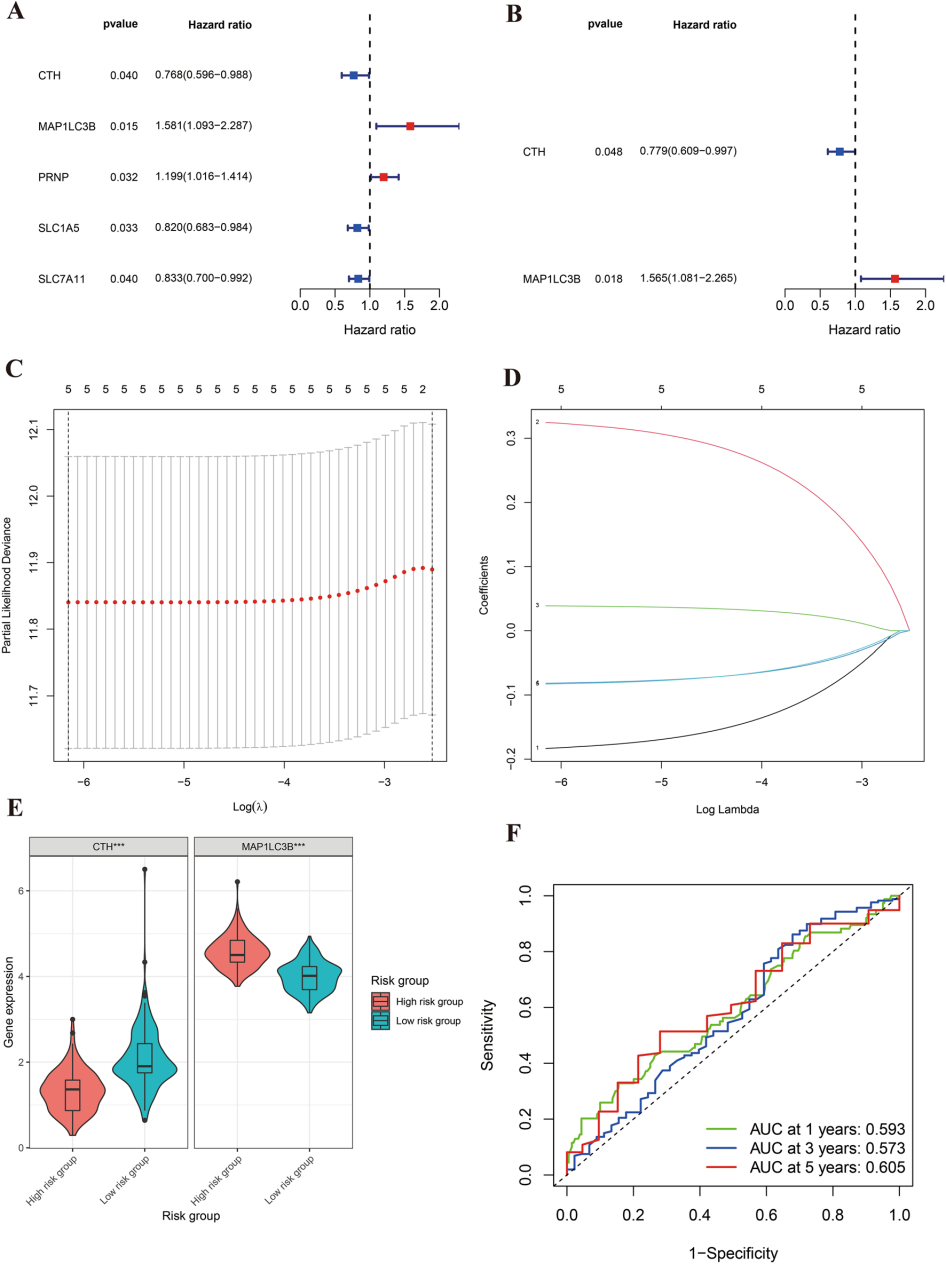
Construction of the Ferroptosis Prognostic Model. Figures (**A**) and (**B**) display univariate and multivariate COX regression analysis results, with red indicating high-risk genes and blue representing low-risk genes. Figures (**C**) and (**D**) illustrate the outcomes of LASSO regression analysis. Figure (**E**) highlights differential expression of CTH and MAP1LC3B in high- and low-risk groups, with "***" signifying p < 0.001. Figure (**F**) shows the ROC diagnostic curve.

Our initial phase in constructing a prognostic model for gastric cancer (GC) ferroptosis involved a univariate Cox regression analysis. This rigorous statistical approach allowed us to narrow down our focus from the broader gene set to five pivotal genes, as depicted in Fig. 4A. This selection provided an initial understanding of the genes potentially playing a significant role in GC ferroptosis.

The results of the univariate and multivariate Cox regression analyses are presented in Fig. 4A, B.

The analysis was then further refined using the Least Absolute Shrinkage and Selection Operator (LASSO) regression technique (Fig. 4C,D). This method, renowned for its efficacy in enhancing model accuracy and preventing overfitting, was instrumental in distilling our findings down to two critical genes: Cystathionine Gamma-Lyase (CTH) and Microtubule Associated Protein 1 Light Chain 3 Beta (MAP1LC3B).

Subsequently, we applied a risk score categorization to our patient cohort, dividing them into two distinct groups based on their calculated risk scores. This categorization revealed 168 high-risk cases and 181 low-risk cases. The delineation of these groups marks a significant step towards understanding the prognostic implications of ferroptosis in gastric cancer and tailoring patient-specific therapeutic approaches.

### Expression of model genes and risk assessment

In an intriguing turn of findings, our analysis revealed a differential expression pattern of the two key genes in the high-risk group. Cystathionine Gamma-Lyase (CTH) exhibited a significantly lower expression in this group (p < 0.001), suggesting its potential role as a protective factor in gastric cancer (GC) progression. This observation is depicted in Fig. 4E. Conversely, Microtubule Associated Protein 1 Light Chain 3 Beta (MAP1LC3B) presented an elevated expression in the high-risk group (p < 0.001), indicating its possible involvement in exacerbating the disease severity.

**Figure 5:**
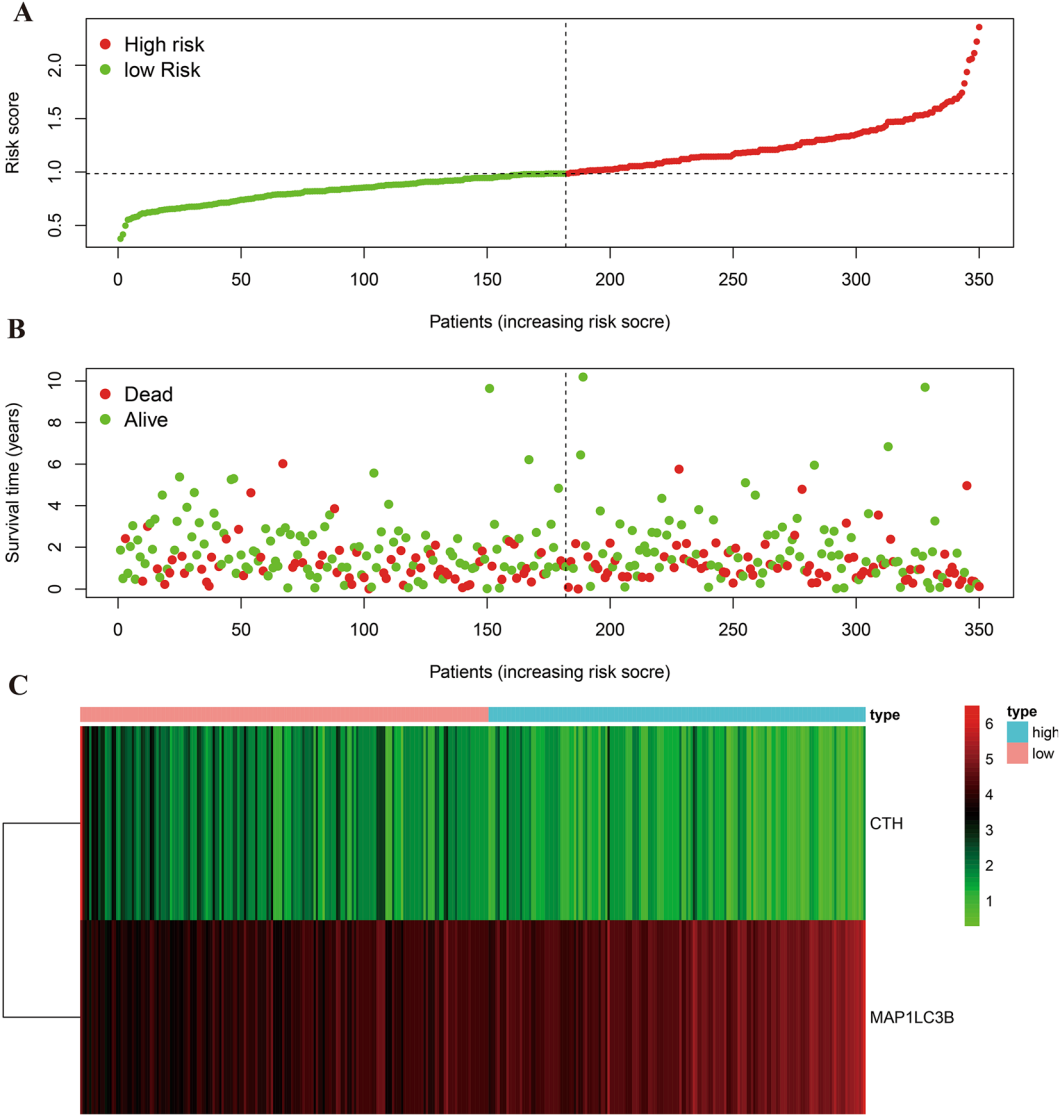
Risk assessment diagram. Figure (**A**) arranges cases according to ascending risk values. Figure (**B**) shows the survival status of each case, and Figure C details CTH and MAP1LC3B gene expression in high and low-risk groups.

Furthermore, our comprehensive risk assessment across all GC cases unveiled a discernible correlation between higher risk scores and increased mortality. This correlation is not only statistically significant but also clinically relevant, as shown in Fig. 5A–C. These results underscore the potential of these genes as prognostic markers in GC and their role in predicting patient outcomes, paving the way for more personalized and targeted therapeutic strategies.

### Survival analysis results

**Figure 6:**
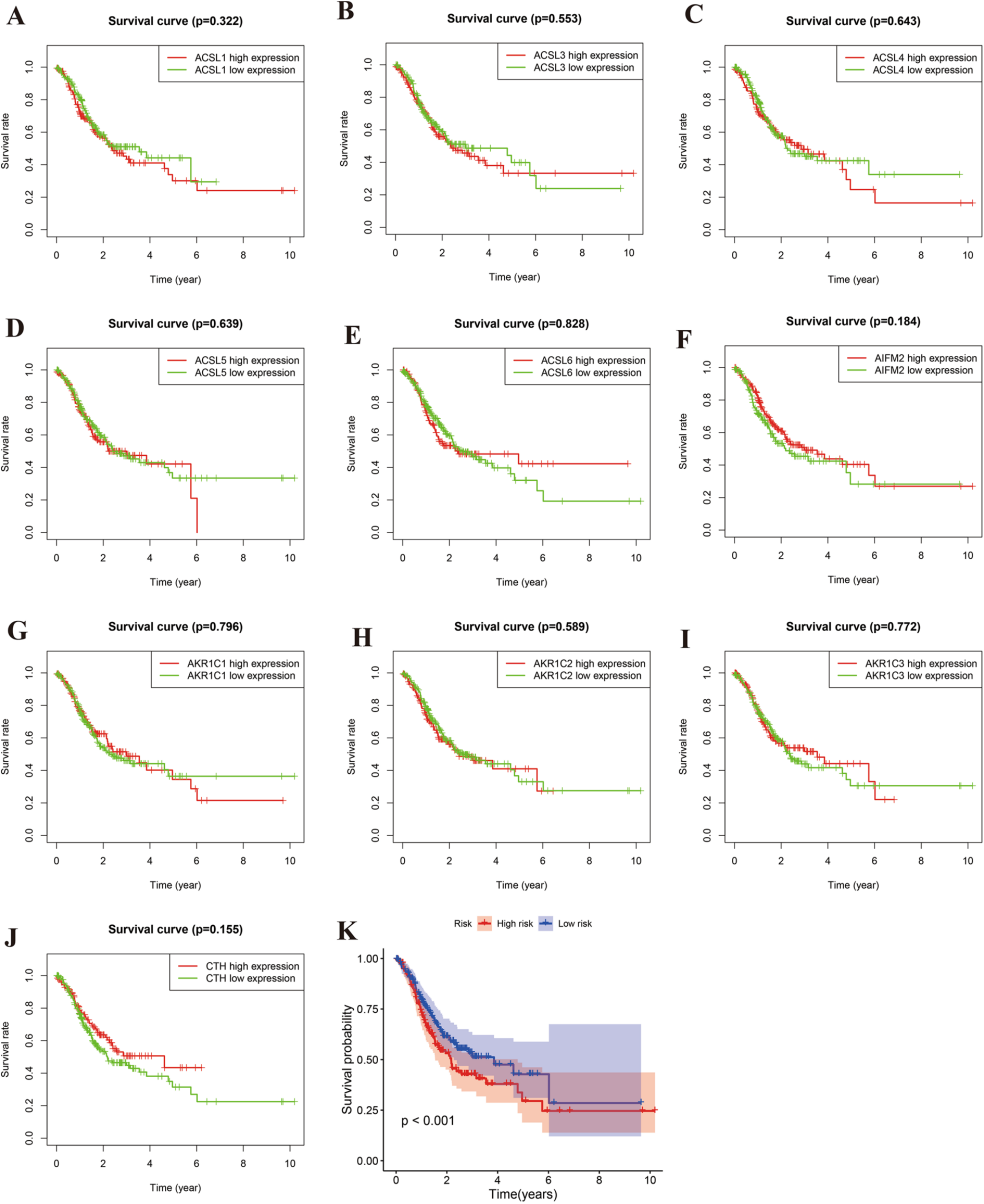
Survival analysis. Figures (**A**–**J**) represent survival curves of GC cases based on individual gene expression. Figure (**K**) contrasts survival rates in high and low-risk groups derived from the prognostic model.

In our comprehensive survival analysis, we embarked on an investigation into the impact of individual gene expressions on the survival outcomes of gastric cancer (GC) patients. This analysis involved grouping GC cases based on the expression levels of each gene. Remarkably, this approach did not yield any significant differences in survival curves, as comprehensively illustrated in Fig. 6A–J. This finding suggests that the expression of these genes in isolation may not be a definitive indicator of survival in GC.

In stark contrast, when we applied our ferroptosis prognostic model, a profound observation emerged. The model delineated a stark difference in survival rates between different risk groups. Notably, the high-risk group, as classified by our model, exhibited significantly lower survival rates, a result bearing statistical significance (p < 0.001). This pivotal finding is displayed in Fig. 6K, highlighting the robustness of our prognostic model in predicting survival outcomes in GC. This result underscores the potential utility of our model in clinical settings, offering a powerful tool for risk stratification and guiding treatment decisions.

### Testing and prognosis prediction of the GC prognostic model

A critical assessment of the gastric cancer (GC) prognostic model was conducted through Receiver Operating Characteristic (ROC) curve analysis. This analysis served as a benchmark to evaluate the model's predictive prowess. The results confirmed the model's relative accuracy, with the area under the ROC curve (AUC) for 1-year, 3-year, and 5-year survival predictions all surpassing the 0.5 threshold, indicative of a model with strong discriminative ability. These findings are clearly represented in Fig. 4F.

**Figure 7:**
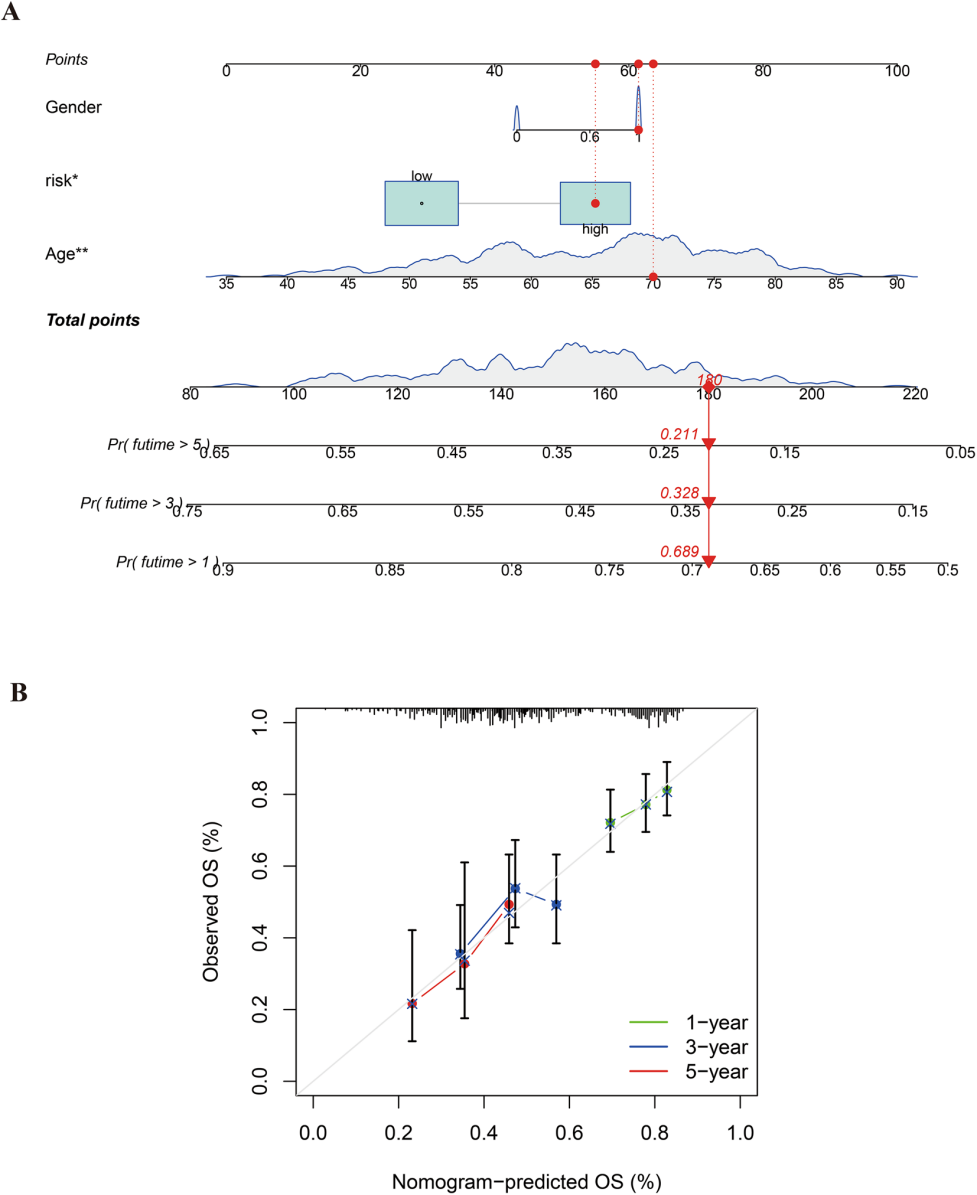
Column line and calibration plots. Figure (**A**) depicts a predicted prognosis column line plot. Figure (**B**) illustrates a calibration plot comparing predicted outcomes with actual scenarios.

Further validation of the model's utility was achieved through the construction of column plots. These plots provided a visual representation of survival probability, taking into account patient-specific baseline information and calculated risk scores, as depicted in Fig. 7A. This approach allowed for an intuitive understanding of the model's practical application in predicting patient outcomes.

Moreover, the model's predictive accuracy was substantiated by calibration curves, as shown in Fig. 7B. These curves demonstrated a close alignment between the predicted and observed survival outcomes, further solidifying the model's credibility and potential utility in a clinical setting for prognostic assessment in GC.

### Drug sensitivity analysis of CTH and MAP1LC3B

A pivotal aspect of our study involved conducting a sensitivity analysis to explore the therapeutic implications of Cystathionine Gamma-Lyase (CTH) and Microtubule Associated Protein 1 Light Chain 3 Beta (MAP1LC3B) in the context of drug responsiveness. This analysis revealed significant associations of these two genes with multiple drug sensitivities, underscoring their potential as key mediators in the treatment of gastric cancer (GC).

**Figure 8:**
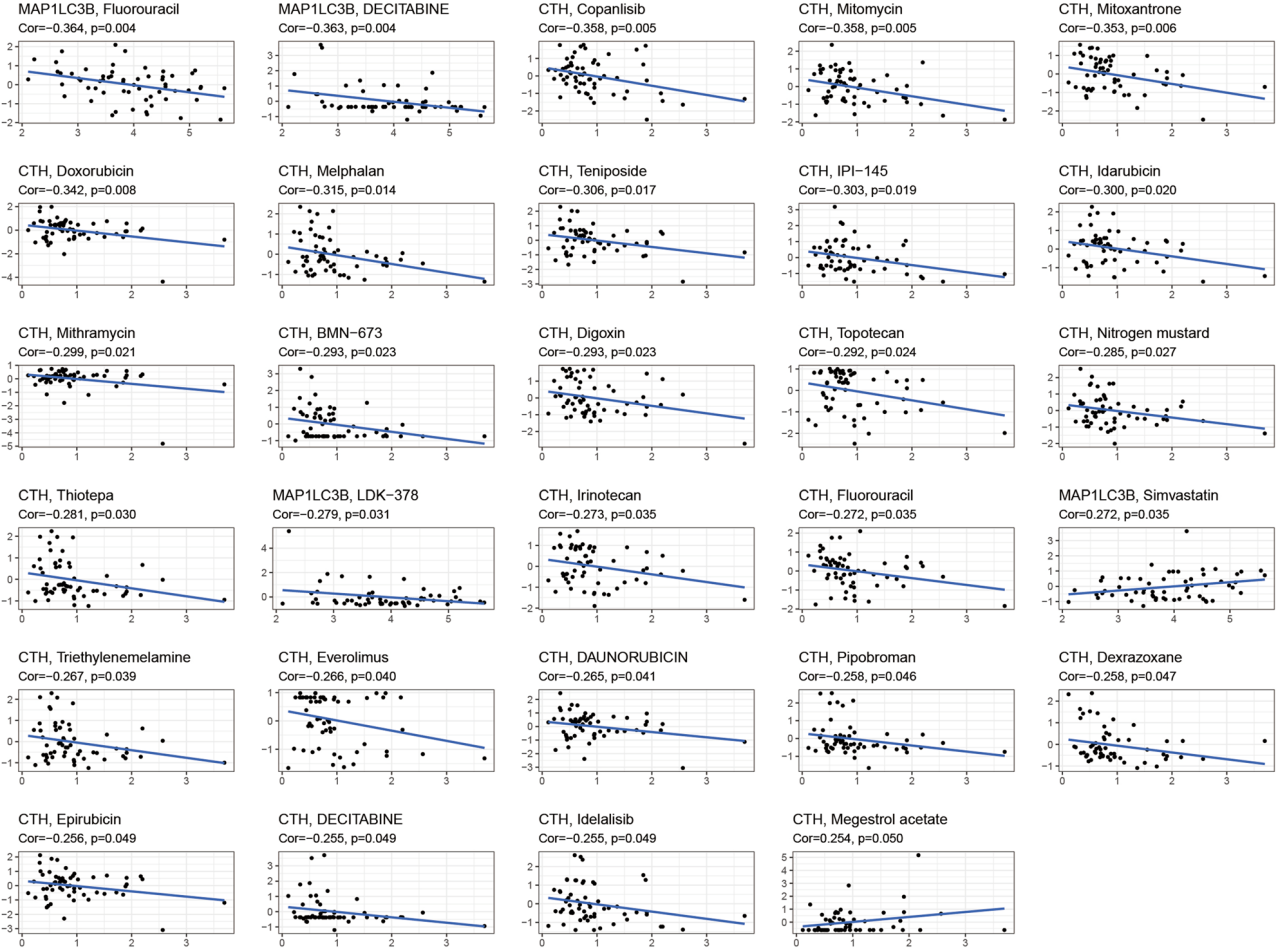
Drug sensitivity plot. This graph displays the drug sensitivity correlation of CTH and MAP1LC3B, where cor > 0 indicates positive correlation and cor < 0 signifies negative correlation with drug sensitivity.

As illustrated in Fig. 8, we observed that CTH and MAP1LC3B exhibited distinct patterns of correlation with various pharmacological agents. Notably, certain drugs demonstrated a positive correlation, suggesting enhanced effectiveness in the presence of higher expression levels of these genes. Conversely, other drugs showed a negative correlation, indicating a possible reduction in efficacy correlating with increased gene expression.

These findings provide valuable insights into the complex interplay between gene expression and drug response in GC. The differential drug sensitivities associated with CTH and MAP1LC3B highlight their potential as biomarkers for personalized treatment strategies, offering a promising avenue for improving therapeutic outcomes in GC patients.

### Immune cell composition and correlation analysis

**Figure 9:**
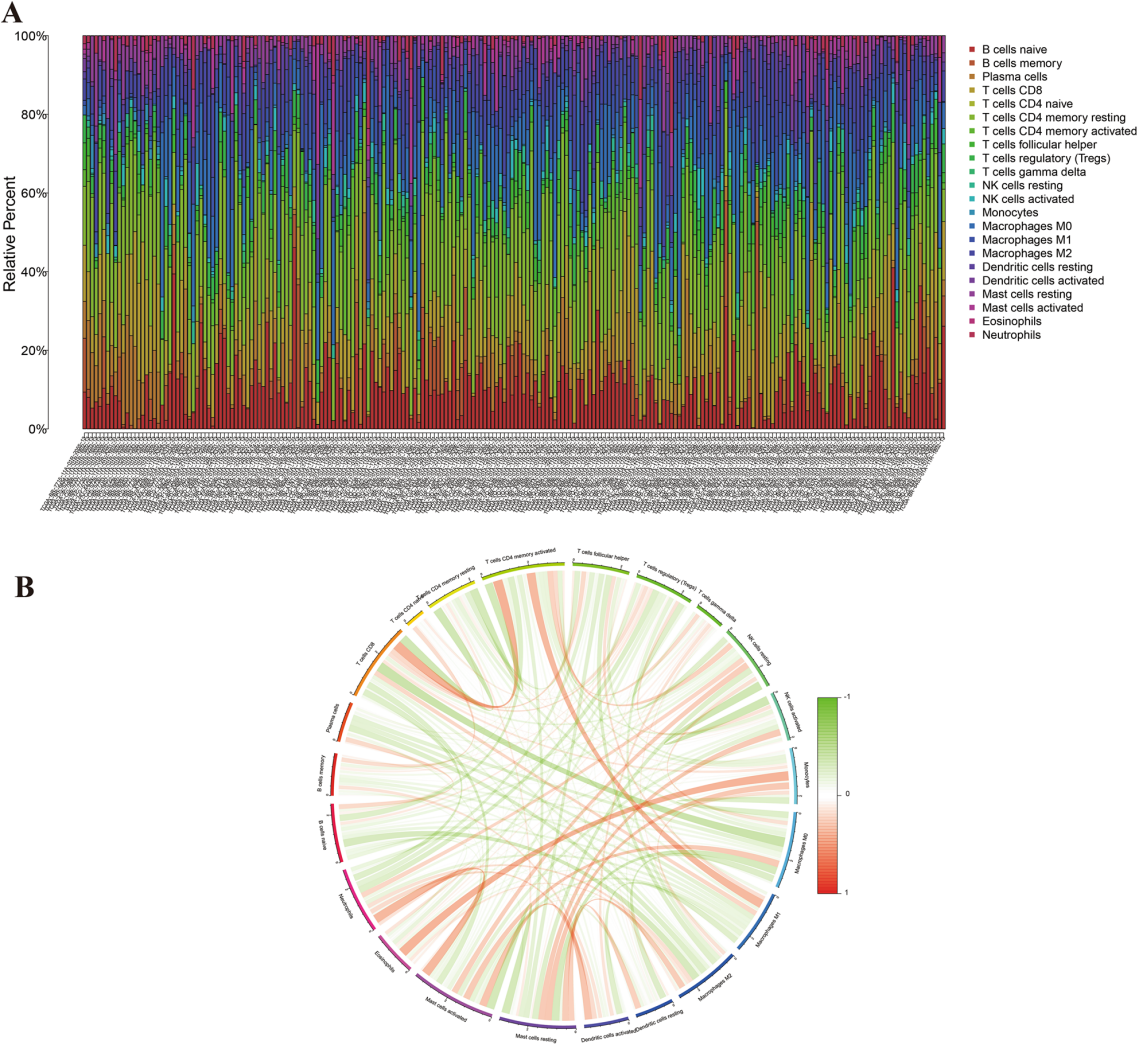
Immune cell composition and correlation analysis. Figure (**A**) shows the immune cell composition in GC and normal cases. Figure (**B**) presents the correlation analysis between immune cell subtypes, with red lines indicating synergistic high expression and green lines suggesting synergistic low expression.

In our endeavor to dissect the immune landscape within gastric cancer (GC), we leveraged the advanced capabilities of the CIBERSORT software. This computational tool enabled us to meticulously analyze and quantify the expression of 22 distinct immune cell subtypes within each GC case. The intricate details of these immune cell profiles are presented in Fig. 9A, offering a comprehensive view of the immune microenvironment in GC.

Beyond mere quantification, our study delved into the complex interactions among these immune cell subtypes. Employing sophisticated correlation analysis, we unveiled a network of both synergistic (positive) and antagonistic (negative) interactions within this milieu. These interactions are intricately mapped out in Fig. 9B, providing a nuanced understanding of the immune cell dynamics in GC. The interplay of these immune cells, revealed through our analysis, underscores the complexity of the tumor microenvironment and its potential implications for GC pathogenesis and treatment.

### Differential and correlation analysis of immune cells in relation to CTH and MAP1LC3B

**Figure 10:**
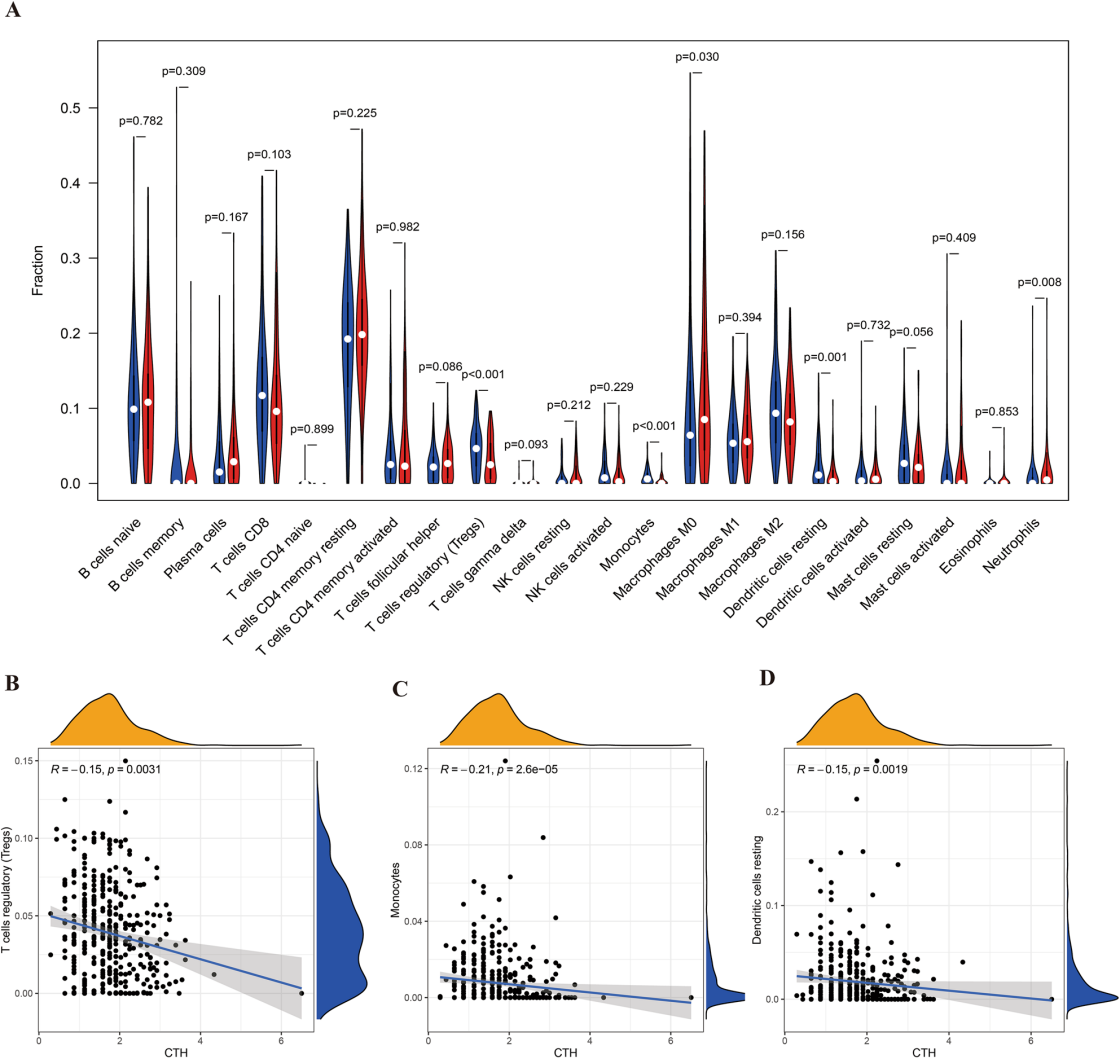
Relationship between CTH and immune cell subtypes. Figure (**A**) visualizes differential analysis between CTH and immune cell subtypes. Figures (**B**–**D**) focus on correlation analysis with these cell types.

**Figure 11:**
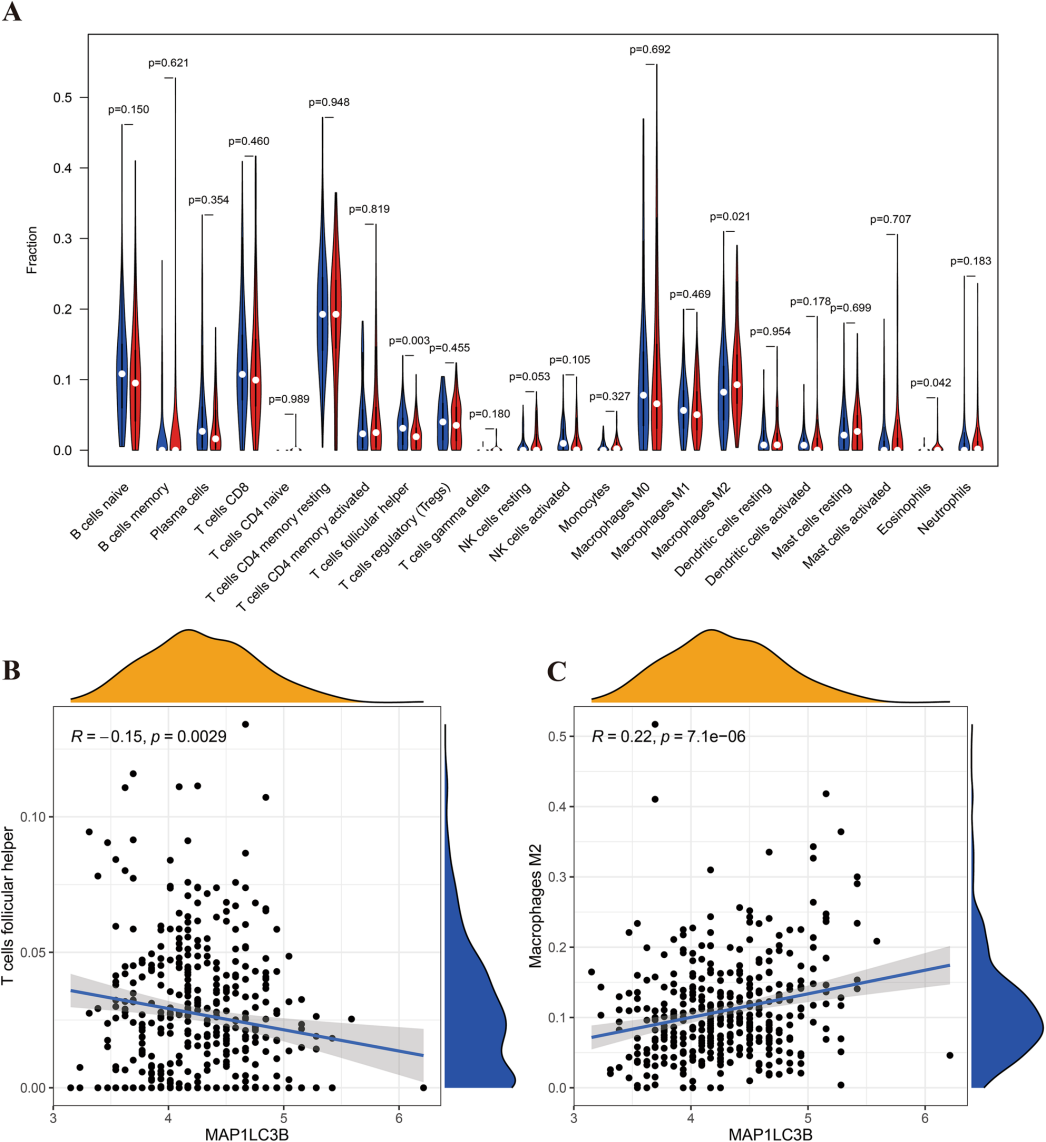
Relationship between MAP1LC3B and immune cell subtypes. Figure (**A**) displays differential analysis between MAP1LC3B and immune cell subtypes. Figures (**B**) and (**C**) focus on correlation analysis with these cell types.

In a further extension of our investigation into the immune microenvironment of gastric cancer (GC), we focused on understanding how variations in Cystathionine Gamma-Lyase (CTH) and Microtubule Associated Protein 1 Light Chain 3 Beta (MAP1LC3B) expressions correlate with changes in immune cell subtypes. Our analysis, as depicted in Figs. 10A–D and 11A–C, revealed significant differential expressions of various immune cell subtypes in relation to the levels of CTH and MAP1LC3B.

A notable aspect of our findings was the negative correlation of CTH with key immune cells, including T regulatory cells (Tregs), Monocytes, and Resting Dendritic cells. This suggests a potential inhibitory role of CTH on these immune cell types, which could have profound implications for the immune response in GC.

Conversely, MAP1LC3B exhibited a diverse range of correlations with immune cells. Particularly, its varying associations with T follicular helper cells and M2 Macrophages were observed, indicating a complex and nuanced role of MAP1LC3B in modulating the immune landscape of GC.

These insights into the relationship between key ferroptosis-related genes and the immune cell milieu not only enrich our understanding of GC pathophysiology but also open up new avenues for exploring targeted immunotherapies in GC treatment.

### Immunohistochemical analysis

**Figure 12:**
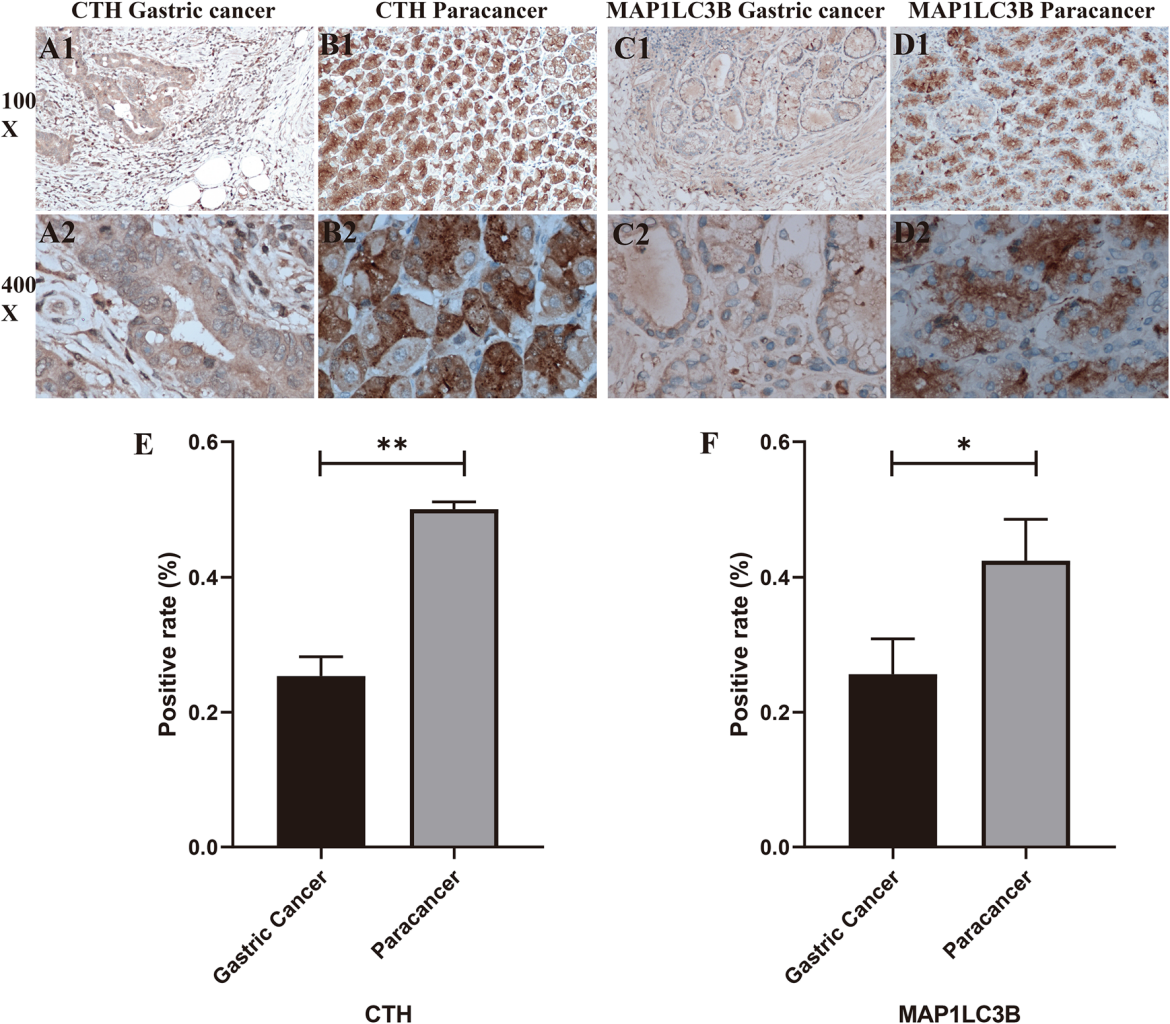
Immunohistochemistry and positive rate statistics. Figures (**A1**–**D2**) present CTH and MAP1LC3B expression in GC and control groups, respectively. Figures E and F summarize positive rate statistics for immunohistochemistry, indicating lower expression of both CTH and MAP1LC3B in GC compared to control groups.

Our immunohistochemical investigations provided a vivid window into the molecular underpinnings of gastric cancer (GC). Utilizing specific staining techniques, we meticulously analyzed six tissue sections each for Cystathionine Gamma-Lyase (CTH) and Microtubule Associated Protein 1 Light Chain 3 Beta (MAP1LC3B). A striking finding emerged from this analysis: both CTH and MAP1LC3B demonstrated markedly lower expression levels in GC tissues when compared to control samples, as evidenced by the images in Fig. 12A1–D2. This reduced expression in GC tissues was congruent with our bioinformatics predictions, reinforcing the validity of our earlier findings. The immunohistochemical positivity rates were calculated using Image J software, and the results are shown in Fig. 12E and F. There were significant differences in the positivity rates of both genes between the experimental group and the control group, with p < 0.05.

**Figure 13:**
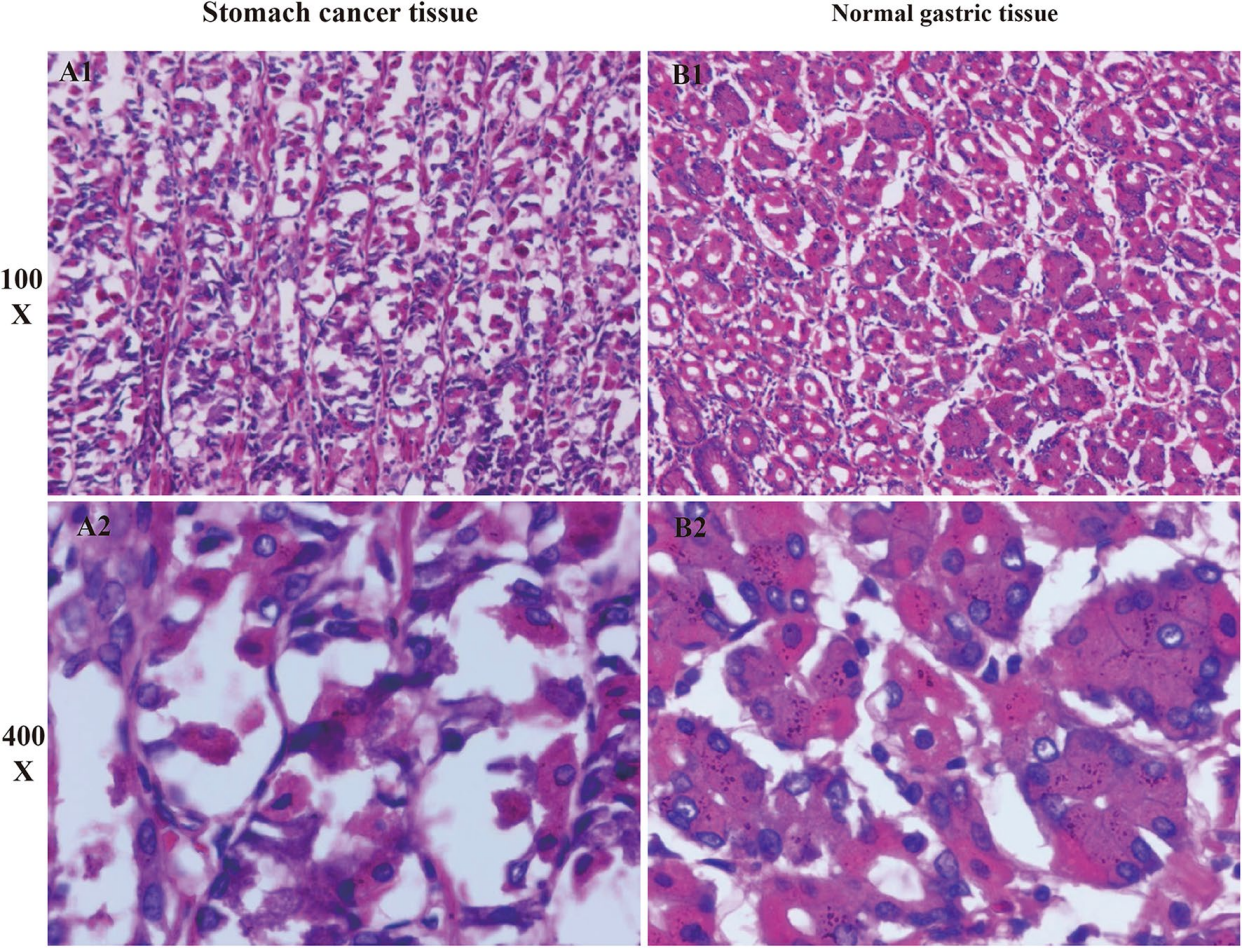
HE staining results. The figures show HE staining of gastric cancer tissue with disrupted nuclear distribution, in contrast to the uniform nuclear distribution in normal gastric tissue (**A1**–**B2**).

Further, our study extended to Hematoxylin–Eosin (HE) staining, which unveiled another layer of GC pathology. The HE-stained slides revealed gastric cancer cells characterized by larger, irregular shapes, along with abnormal chromatin and nuclear features, as detailed in Fig. 13A1–B2. These morphological alterations provide a deeper understanding of the cellular changes inherent in GC and complement our findings on the molecular level.

## Discussions

Our study delved into the intricacies of Ferroptosis genes and revealed initial Gene Ontology (GO) enrichment primarily centered around cellular transition metal ion and cellular iron ion homeostasis. The KEGG pathway analysis underscored enrichment in Ferroptosis, Mitophagy in animals, Chemical carcinogenesis—reactive, and Fatty acid degradation. This aligns with existing knowledge that lipid peroxide accumulation is crucial for Ferroptosis initiation. Activated ACSL4, by catalyzing the synthesis of lipids containing polyunsaturated fatty acids, contributes to the build-up of lipid peroxidation products, culminating in iron-dependent Ferroptosis^29^. Additionally, polyunsaturated fatty acids selectively induce Ferroptosis in cancer cells within acidotic environments^30^. Ferroptosis is implicated in diverse pathological states, including cancer, infections, ischemic tissue damage, and neurodegenerative diseases^31^. Furthermore, correlations between hypercholesterolemia, dyslipidemia, and increased cancer risk are well-documented. These conditions affect cancer pathogenesis by promoting resistance to anti-ferritinase cell death^32^. Increasing evidence supports the occurrence of Ferroptosis in various tumors^33–36^. which echoes our findings. Our analysis indicates that genes differentially expressed in GC show significant enrichment in the Ferroptosis pathway, potentially contributing to the poor prognosis of GC patients.

Studies have demonstrated the critical importance of understanding the regulatory networks within complex biological systems through theoretical and computational models for the impact on disease progression and treatment responses. The identification of potential therapeutic targets via simulation and analysis of specific signal transduction pathways holds significant implications for designing new therapies for complex diseases such as cancer^37^. Additionally, research has shown that applying physical and mathematical models to predict and validate the internal interactions of biological systems can reveal the subtle mechanisms controlling intracellular signal transduction. This interdisciplinary approach is crucial for understanding disease development and informing treatment strategies^38^.

Cystathionine Gamma-Lyase (CTH), a protein-coding gene, is primarily linked to hyperhomocysteinemia and cystathionuria. Recent research highlights that IL1 receptor accessory proteins can mitigate apoptosis in Ewing's sarcoma by enhancing the activity of the Xc-cystine/glutamate antiporter and CTH transcription, thereby maintaining cysteine levels for reactive oxygen species detoxification^39^. Notably, CTH has been implicated in triple-negative breast cancer (TNBC). Studies show that radiation-resistant TNBC cells undergo constitutive phosphorylation of eIF2α, activating ATF4 and upregulating genes associated with glutathione biosynthesis, such as CTH, GCLC, and SLC7A11. This process increases intracellular levels of reduced glutathione (GSH), which is crucial in TNBC development^40^. Intriguingly, in our study, the prognostic models incorporating CTH indicated markedly lower survival rates in high-risk groups. CTH's influence extends to the modulation of T cell regulatory (Tregs), Monocytes, and resting Dendritic cell immune subtypes.

Moreover, studies suggest that the microflora can modulate intra-tumor monocytes, thereby fostering antagonistic tumor immune responses^41^, a finding corroborated by our research. Our study further reveals that Monocytes are significantly dysregulated in GC. This dysregulation may lead to alterations in the tumor's immune microenvironment, potentially resulting in diminished tumor engraftment, thereby facilitating tumor cell growth and contributing to a poorer prognosis in GC.

Microtubule Associated Protein 1 Light Chain 3 Beta (MAP1LC3B) is linked with conditions such as Hermansky-Pudlak Syndrome and Granulomatous Disease. In a groundbreaking study, Burcu Aykac Fas et al. scrutinized the impact of 26 missense mutations in MAP1LC3B/LC3B from pan-cancer analyses. They discovered that damaging or neutral mutations in this ubiquitin-like protein are pivotal in cancer development^42^. Remarkably, emerging research suggests that the molecular mechanism of autophagy augments Ferroptosis by selectively targeting anti-ferroptosis regulators, with MAP1LC3B playing a significant role in this process^43^. This finding aligns with our research outcomes. Our study revealed that GC patients in the high-risk group, as determined by the Ferroptosis prognostic model incorporating CTH and MAP1LC3B, exhibited substantially lower survival rates compared to those in the low-risk group. Furthermore, MAP1LC3B exhibited a negative correlation with M2 Macrophages.

It has been reported that histamine and its receptor HRH1, often upregulated in the tumor microenvironment, can induce T cell dysfunction. HRH1 also promotes macrophage differentiation towards M2-like immunosuppressive phenotypes, enhancing immune checkpoint VISTA expression and leading to T cell dysfunction^44^. Intriguingly, these observations are in harmony with our study's findings. Our research also indicates that M2 macrophage dysregulation in GC may contribute to altered tumor microenvironments, facilitating tumor cell growth in the absence of immune cell inhibition.

The innovative aspect of my study's methodology includes a strategic use of univariate Cox regression for initial gene screening in gastric cancer, which is then refined through multivariate Cox regression to determine independent effects. The incorporation of LASSO regression for variable selection adds to the robustness and specificity of the prognostic model. This combination of methods offers a more detailed and precise approach to understanding the role of ferroptosis in gastric cancer, setting it apart in the field.

Future research should focus on validating our algorithm across diverse gastric cancer populations and in different clinical settings. Additionally, incorporating emerging genomic data and advanced analytical techniques could further enhance our model’s predictive power. A key limitation of our current approach is its dependency on existing datasets, which may not encompass the full genetic diversity of gastric cancer. Addressing these aspects in future work will be crucial for improving the prognostic accuracy and clinical utility of our findings.

In our study, we meticulously analyzed 407 cases, integrating genome-wide expression and clinical data of gastric cancer (GC) to develop a Ferroptosis-related prognostic model. Our findings identified significant downregulation of CTH and MAP1LC3B in GC, corroborated by immunohistochemical methods. Additionally, we observed a notable dysregulation of monocyte-macrophage dynamics in GC, suggesting their potential role in the progression of the disease.

## Conclusions

The dysregulation of Cystathionine Gamma-Lyase (CTH), Microtubule Associated Protein 1 Light Chain 3 Beta (MAP1LC3B), and monocyte-macrophage dynamics are critical determinants of the poor prognosis associated with gastric cancer (GC). Our findings suggest that CTH and MAP1LC3B hold potential as prognostic biomarkers for GC, offering valuable insights to enhance early diagnosis and inform treatment strategies.

## Data Availability

The datasets supporting the conclusions of this article are available in the TCGA database (https://portal.gdc.cancer.gov/).
